# Heightened IDO1 levels predict Bacillus Calmette-Guèrin failure in high-risk non-muscle-invasive bladder cancer patients

**DOI:** 10.1038/s41420-025-02489-7

**Published:** 2025-04-26

**Authors:** Alice Turdo, Gabriele Tulone, Sebastiano Di Bella, Gaetana Porcelli, Caterina D’Accardo, Miriam Gaggianesi, Chiara Modica, Simone Di Franco, Francesca Angeloro, Giulia Bozzari, Vincenzo Davide Pantina, Melania Lo Iacono, Cristina Minasola, Rosa Giaimo, Anna Martorana, Nicola Pavan, Matilde Todaro, Alchiede Simonato, Giorgio Stassi

**Affiliations:** 1 Department of Health Promotion, Mother and Child Care, Internal Medicine and Medical Specialties, University of Palermo, Palermo, Italy; 2 Department of Precision Medicine in Medical, Surgical, and Critical Areas, University of Palermo, Palermo, Italy; 3 Azienda Ospedaliera Universitaria Policlinico (AOUP) “Paolo Giaccone”, Palermo, Italy

**Keywords:** Biomarkers, Cancer

## Abstract

Recent studies have indicated a potential link between immune-related gene expression and Bacillus Calmette-Guèrin (BCG) treatment response in non-muscle-invasive bladder cancer (NMIBC) patients, however, prognostic gene signatures have not significantly improved risk stratification beyond clinical characteristics. To identify predictive biomarkers in T1 high-risk (HR) bladder cancer (BC) patients responding to BCG treatment, a gene signature was derived from a discovery cohort of 73 BCG-naïve patients, both responders and non-responders, using the publicly available dataset GSE1542618. Among the identified genes, Indoleamine 2,3-dioxygenase (IDO1), an immunosuppressive enzyme, emerged as a crucial determinant of treatment outcomes. The association between IDO1 expression and worse prognosis was subsequently validated in a cohort of 75 BC patients using formalin-fixed paraffin-embedded (FFPE) BC specimens collected prior BCG treatment. This research revealed significant insights into the mechanisms underlying unsatisfactory responses to BCG treatment in HR patients, posing IDO1 as a promising prognostic biomarker and therapeutic target for NMIBC.

## Introduction

Bladder cancer (BC), the ninth most common cancer worldwide, comprises a non-muscle-invasive (NMIBC) and muscle-invasive (MIBC) form. NMIBCs account for 75% of BCs, with 10–15% of these cases progressing to the more severe MIBC. Therapeutic approaches vary based on risk stratification: low-risk NMIBC patients typically undergo transurethral resection (TURBT) alone, while intermediate- and high-risk patients often receive adjuvant treatments to curtail disease recurrence and progression [1].

For high-risk (HR) NMIBC patients post-TURBT, the gold standard adjuvant therapy involves intravesical instillation of Bacillus Calmette-Guérin (BCG). BCG treatment uses a live-attenuated mycobacterium tuberculosis, which induces both innate and acquired immune responses in BC patients. Although the precise mechanism of action remains unclear, BCG is believed to have direct effects on cancer cells, including the activation of apoptosis and oxidative stress response [2].

Numerous meta-analyses have demonstrated that BCG therapy effectively reduces recurrence rates and delays disease progression [3, 4]. However, a significant subset of patients fails to respond to intravesical BCG due to intolerance, refractoriness, or relapse. Studies have shown that within 36 months of therapy, 20% of BCG patients experience side effects leading to intolerance. Moreover, up to 40% of patients face recurrence or relapse after a 6-month disease-free period. These challenges highlight the need for improved treatment strategies and patient selection methods for BCG therapy in NMIBC management [5, 6].

The European Association of Urology (EAU) guidelines provide a comprehensive definition for “BCG-unresponsive” tumors in BC. This classification encompasses BCG-refractory tumors and recurrent T1/Ta high-risk (HR) tumors. Tumors are considered BCG-unresponsive if they recur within 6 months of completing adequate BCG exposure or it is detected the presence of carcinoma in situ (CIS) within 12 months. “Adequate BCG exposure” is determined by the completion of at least five out of six doses of a first induction course, plus a minimum of two out of six doses of a second induction course or at least two out of three doses of a maintenance regimen [7].

This subgroup of patients, who do not respond to BCG therapy, are candidates for radical cystectomy. However, the delays caused by multiple instillation cycles lead to significant disease progression in some cases, further worsening their prognosis.

Several studies have been conducted to identify clinical factors and biomarkers that could predict therapy response [5]. Several risk factors have been associated with an increased likelihood of recurrence in BCG patients, including female gender, age over 70 years old, overweight and obesity, a preoperative neutrophil to lymphocyte ratio exceeding 2.5 and high heaviness of smoking index [8]. In addition to clinical factors, researchers have explored various molecular and microbiological aspects that might influence BCG therapy response. The tumor mutational burden, neoantigen load, and mutations in DNA damage response genes within BC cells have been suggested as potential indicators of treatment efficacy [9]. Another emerging highly debated field of interest is the association between response to BCG therapy and the bladder microbiome, which may modify the immune repertoire of the urinary tract toward an immunosuppressive pattern [10]. Recent studies have also highlighted the role of immune cell phenotypes in treatment outcomes. An elevated T-cell exhaustion phenotype (CD8^+^PD-1^+^) has been correlated with treatment failure and patient relapse in BC [11]. Based on these data, numerous immune checkpoints inhibitors have been prompted in clinical settings with a limited efficacy [12], underlying the need of a more comprehensive understanding of the immune mechanisms involved in the treatment response of BC.

Despite extensive research efforts aimed at uncovering the mechanisms of susceptibility or resistance to BCG treatment in BC, a significant gap remains in the ability to predict therapy response with high sensitivity and specificity. To address this challenge, our study employed a comprehensive multiomics analysis, examining the molecular profiles of both responsive and unresponsive BC patients treated with BCG. Our analysis led to the identification of a set of differentially expressed genes, which among these, the enzyme Indoleamine 2,3-dioxygenase (IDO1) emerged as a particularly promising predictive biomarker for BCG therapy response. This finding has the potential to provide clinicians with a valuable tool for assessing the likelihood of treatment success in individual patients.

## Results

### BCG therapy failure is dictated by the immune system response in T1 HR NMIBCs

Intravesical administration of Bacillus Calmette-Guérin (BCG) is the recommended first-line adjuvant immunotherapy for patients with NMIBCs. Approximately half of the patients who undergo BCG therapy fail to respond adequately, and alarmingly, one-fifth of cases progress to MIBC [1]. Therefore, predicting the response to BCG therapy represents a significant clinical challenge in the management of BC, enhancing therapeutic decision-making processes.

**Fig. 1:**
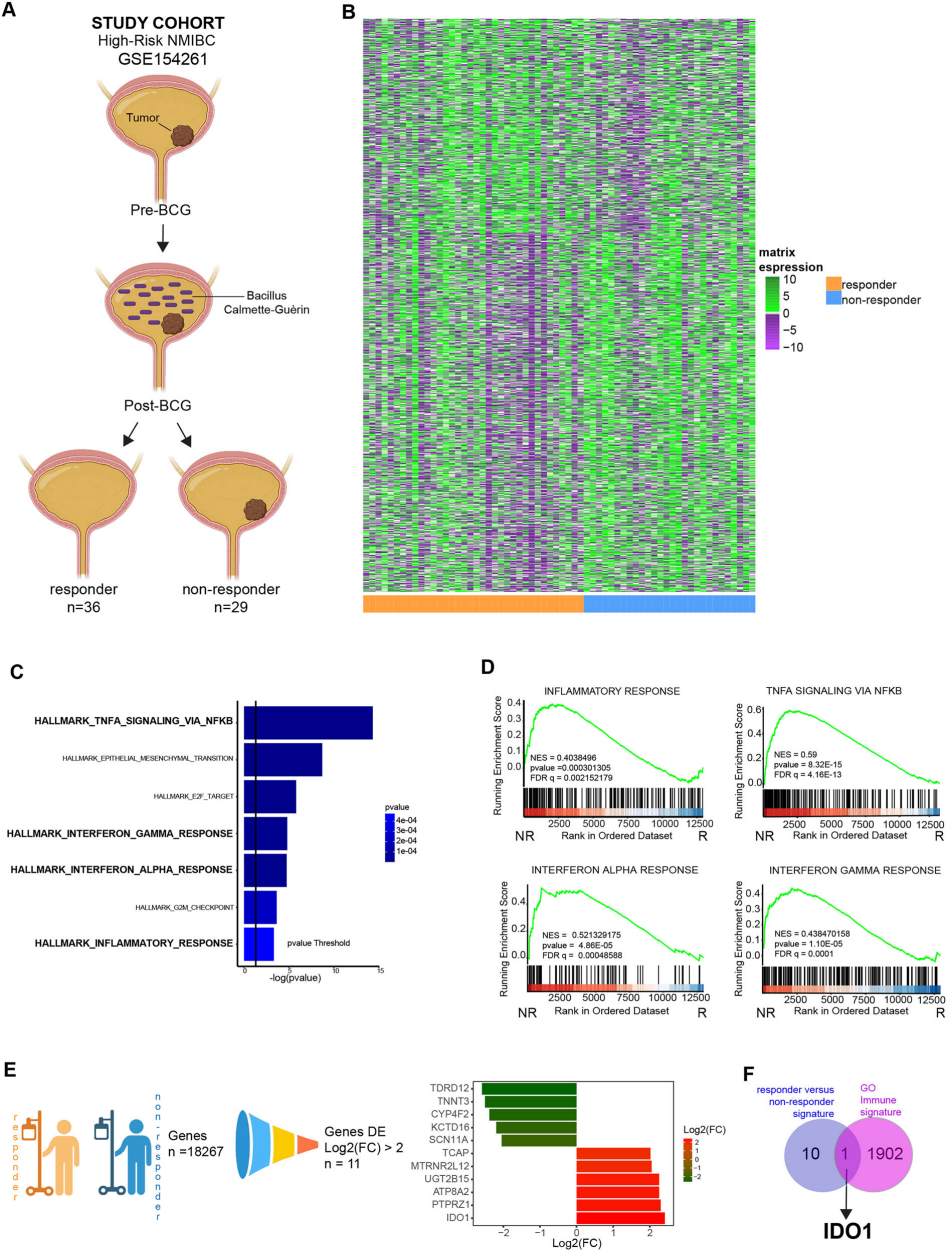
Gene expression analysis of a validation cohort of BC patients highlighted a gene expression signature associated with BCG therapy response. **A** Workflow chart indicating the process to select naïve BC patients according to the response to BCG treatment, retrieved from GSE154261 database (discovery cohort). **B** Heatmap of differential expressed genes (DEGs) performed with the R edgeR library in responder versus non-responder BC patients (*p*-value < 0.05). **C** Enrichment analysis using the EnrichR library in Ontology terms Biological Process, Molecular Function and Cellular Component of DEGs from responder versus non-responder BC patient cohort. **D** GSEA plot performed, between Non-Responder (NR) vs Responder (R), with the MSigDB library in the C2 class for inflammatory response, TNFA signaling via NFKB, Interferon alpha response and Interferon Gamma Response. **E** Funnel graph to filter 18,267 DE Genes, starting from 59,000 genes, 1246 genes have a *p* value < 0.05, and 11 are coding genes with an abs(fc) ≥ 2 (left). Barplot showing 11 top DEGs (right). **F** Venn diagram showing the intersection between 11 top DEGs and genes belonging to the GO immune signature (*n* = 1903).

To identify a predictive gene signature for determining the response to BCG in T1 high-risk (HR) BC patients, we conducted an analysis of RNA-Seq data. Data sourced from 73 naïve BCG samples, which were obtained from the publicly available dataset GSE154261. This comprehensive analysis aimed to uncover biomarkers that could potentially indicate how patients with T1 HR BC might respond to BCG treatment [13] (Fig. 1A).

The training cohort consists of 65 samples, including 36 responders and 29 non-responders (Fig. 1A and Fig. S1A). We utilized the transcriptomic profiles of these groups to conduct a differential gene analysis. Out of nearly 59,000 initial genes, 18,267 genes were retained after performing differential expression analysis using the R edgeR library, with 1246 genes showing a p-value of less than 0.05. Particularly, after conducting unsupervised hierarchical clustering, the patients in the training cohort were dichotomized into responders and non-responders to BCG therapy. This enabled the identification of differentially expressed genes (DEGs) associated with BCG response in NMIBC patients (Fig. 1B).

The enriched top pathways and the gene set enrichment analysis (GSEA) showed a significant modulation of the signaling related to the TNFα/NFκB, the epithelial to mesenchymal transition, the E2F targets, the IFN-γ and IFN-α response, the G2M checkpoint and inflammatory response in patients with the worst overall survival (Fig. 1C, D and Fig. S1B). These data provide evidence that the response to BCG treatment in patients with BC may be related to the expression of genes involved in the cell-mediated immune system response. Interestingly a wider REACTOME pathway analysis confirmed the involvement of a subset of genes associated with the interferon-mediated immune response to the lack of response to BCG therapy but also highlighted the implication of 6 gene signatures related to the HER2 pathway (Fig. S1C, D). HER2 has, in fact, recently been described as an independent factor of BCG failure in NMIBC [14], thus further corroborating the robustness and reliability of our findings.

To deepen the analysis, we focused our attention on the 11 differentially expressed genes that exhibited a fold change superior to 2 in BCG non-responder versus responder patients. These genes include *ATP8A2*, *CYP4F2*, *IDO1, KCTD16*, *MTRNR2L12*, *PTPRZ1*, *SCN11A*, *TCAP*, *TNNT3*, *TDRD12*, *UGT2B15* (Fig. 1E). To contextualize the differentially expressed genes within a broader framework of immune response mechanisms, potentially revealing insights into the biological pathways that influence BCG therapy outcomes, genes were further merged with coding genes from the Gene Ontology (GO) Immune-related gene signature, which comprises a total of 1,903 genes. Among the identified genes, IDO1 emerged as a unique gene significantly associated to BCG therapy failure (Fig. 1F).

### IDO1 expression is associated with clinicopathological BC features

**Fig. 2:**
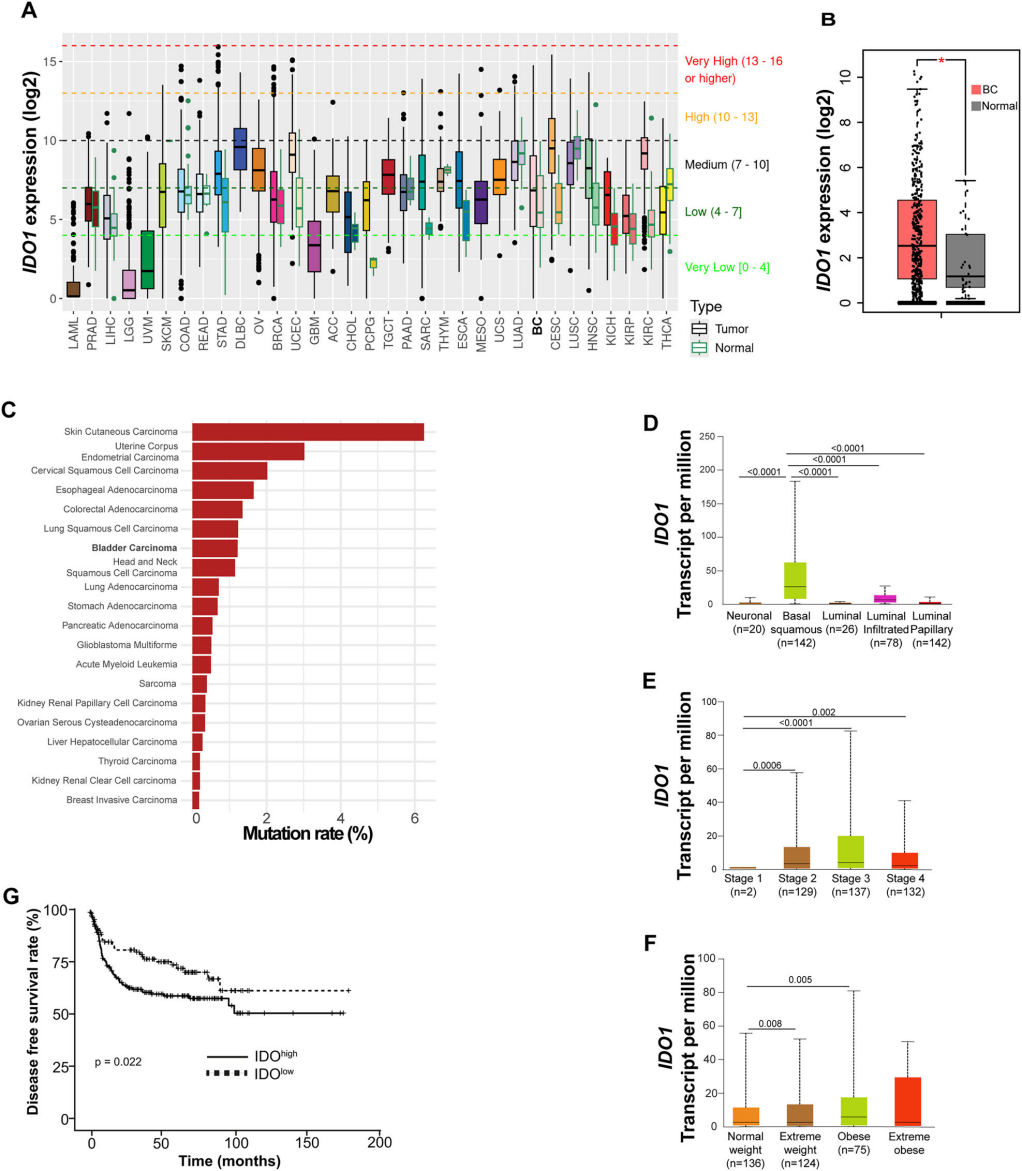
IDO1 expression is negatively associated with BC prognosis. **A** *IDO1* log2 expression in tumor (black box plot frame) versus normal (green box plot frame) samples retrieved from TCGA. Bladder cancer (BC) is shown in bold. **B** IDO1 log2 expression in BC (red box plot) (*n* = 404) and normal tissue (grey box plot) (*n* = 28) retrieved from GEPIA. **C** Percentage of IDO1 mutations for each tumor type sample as in (**A**). (**D-F**) RNA-seq expression data of *IDO1* in normal bladder tissue and in different BC molecular subtypes (**D**), stages (**E**) and weight status (**F**) retrieved from the TCGA database and analyzed by UALCAN. (**G**) Kaplan Meier disease free survival curves of BC patients (GSE32548, GSE48075, GSE31684) stratified by high (*n* = 180) or low (*n* = 117) IDO1 expression levels. Statistical analysis has been performed with log-rank test.

To characterize the role of IDO1 in cancer pathogenesis, the publicly available omics data analysis platform GEPIA and TCGA have been queried. A comprehensive analysis indicated that, in comparison with normal tissue, IDO1 resulted highly expressed in more than twenty tumor types including BC (Fig. 2A). Particularly, IDO1 expression has been significantly associated to BC in *n* = 404 specimens as compared to adjacent -normal or non-tumoral samples (*n* = 28) (Fig. 2B). In cancer compartment, *IDO1* displayed few alterations in approximately twenty different tumors (Fig. 2C and Table S1). These findings indicate that while mutations in the *IDO1* gene itself are infrequent across various tumor types, including BC, the expression and activity of IDO1 are significantly modulated by the surrounding environment. This regulation is crucial for the establishment of a therapy-refractory phenotype in tumors, highlighting IDO1 as a potential target for therapeutic intervention in cancer immunotherapy. Correlation of IDO1 expression with clinicopathological parameters revealed that IDO1 characterizes the two molecular subtypes of BC mostly associated to the expression of the immune checkpoint inhibitors (PD-L1 and CTLA4), basal squamous and luminal infiltrated BC (Fig. 2D). Interestingly, patients with stage 2, stage 3 and the most severe stage 4 BC are characterized by a high expression of IDO1 (Fig. 2E) as well as patients with extreme weight (BMI 24.9–29.9) and obesity (BMI 30–34.9) (Fig. 2F), which have been associated with less favorable outcome in BC patients [15]. Given IDO1 prospective role in regulating disease history, we queried sequencing data by three different cohort of T1 HR BCG-treated BC patients retrieved from GSE32548, GSE48075 and GSE31684 (*n* = 297) for which follow-up data up to 16 years were available. Analysis of survival curves of BC patients showed a significant reduction in disease free survival probability in patients bearing high expression levels of IDO1 (Fig. 2G and Table S2).

These data suggest that higher levels of IDO1 expression correlate with more advanced disease, suggesting its potential role as a marker for tumor progression.

### BCG treatment failure is correlated with defective anti-cancer immune responses

**Fig. 3:**
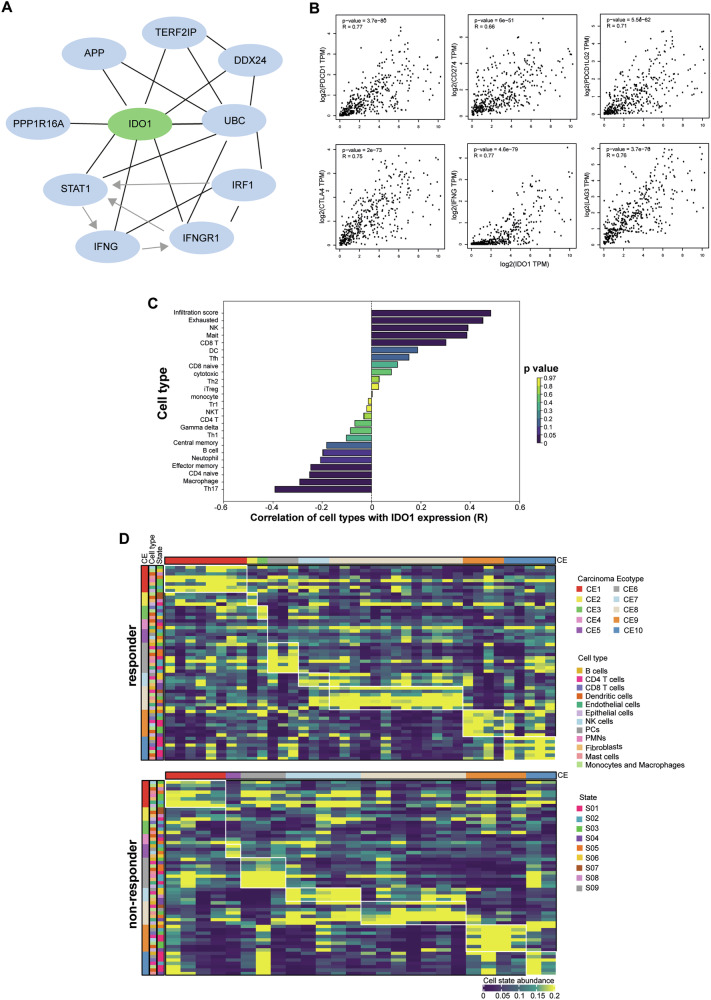
BCG response is correlated with anti-tumor immune response. **A** Functional protein association network of IDO1 based on canSAR.ai database. **B** Scatter plot showing correlation analysis computed on log2 expression data of *IDO1* and *PDCD1*, *CD274*, *CTLA4* or *IFNG*. **C** Correlation between the abundance of immune cells and IDO1 expression in the GSE154261 patients cohort, performed by ImmuneCellAI. *p*-value is indicated by different bar colors. **D** Cell state abundance patterns in responder (*n* = 38) and non-responder (*n* = 26) BC patient cohort (GSE154261), with cell states organized into different cell types and carcinoma ecotypes (CE) according to the EcoTyper machine learning framework [17] and CIBERSORTx analysis [18].

IDO1 plays a significant role in immune evasion mechanisms, which are frequently associated with tumor progression and resistance to therapy. It is an enzyme that catalyzes the breakdown of tryptophan, resulting in the production of kynurenine. This metabolite has been shown to inhibit T-cell proliferation and promote the differentiation of regulatory T-cells. The resulting immune suppression can potentially hinder effective anti-tumor responses during BCG therapy. Recent research has indeed demonstrated a correlation between elevated T cell exhaustion and BCG failure, further supporting the importance of IDO1 in the context of BC treatment outcomes [11]. Pathway analysis data highlighted that *IDO1* transcription activation is regulated by STAT1 pathway, likely under the influence of IFN-γ or as recently demonstrated by the COX- 2/PGE2 axis [16] (Fig. 3A). In accordance, co-expression data revealed that *IDO1* strongly correlated to *PDCD1* (PD-1), *CD274* (PD-L1), *PDCD1LG2* (PD-L2), *LAG3* and *CTLA4* immune checkpoints and IFNG in BC, with a positive correlation coefficient of 0.77, 0.66, 0.71, 0.76, 0.75 and 0.77, predicting that these genes could participate into the immunosuppressive biological process involving T cell dynamics (Fig. 3B). Hence, the interrogation of ImmuneCellAI tool, highlighted that IDO1 may influence T cell disfunction in our discovery cohort of BC patients treated with BCG (Fig. 3C).

To gain deeper insights into the immune landscape of responder versus non-responder bladder tumors, we employed an integrated approach combining the EcoTyper machine learning framework [17] and CIBERSORTx analysis [18]. This comprehensive methodology was applied to the gene expression profiles of the discovery cohort of BC patients (GSE154261), enabling us to construct a high-resolution portrait of the immune microenvironment. The EcoTyper deconvolution process allowed us to characterize diverse multicellular communities, revealing distinct cell type-specific transcriptional programs, which we refer to as cell states. Our analysis yielded significant findings, demonstrating a notably higher abundance of tumor-reactive B cells, CD4, CD8 T cells, and dendritic cells in responder patients compared to their non-responder counterparts (Fig. S2A–C). These results provide valuable insights into the immunological differences between patients who respond to treatment and those who do not, potentially informing future therapeutic strategies and patient stratification approaches. Notably, responder patients exhibited a pronounced increase in activated B cells, as well as naïve and central memory B and T cells, whereas non-responder patients uniquely display the presence of CD4 T regulatory cells (Fig. S2A–D) [17], known to repress tumor-specific CD8 T cells cytotoxicity. Responders’ immune profiles also revealed a higher presence of myeloid dendritic cell subtypes DC1 and DC2 (Fig. S2D) [17], suggesting that these specific dendritic cell populations may play a pivotal role in augmenting T cell anti-tumor activity.

Collectively, our data underscore the importance of T cells as critical determinants of response to immunotherapy, highlighting their integral role within an anti-tumor immune ecosystem. This insight paves the way for future strategies aimed at enhancing therapeutic efficacy in BC treatment.

### IDO1 jeopardizes the responsiveness to BCG therapy in a validation cohort

To validate whether IDO1 expression is associated to BCG response in a validation cohort of T1 HR patients, follow up data and BC samples have been retrieved from a retrospective cohort of *n* = 28 responder and *n* = 47 non-responder BC patients who received six weekly instillations of BCG as induction therapy and successively maintenance therapy at P. Giaccone University hospital (Table 1). Univariate analysis of clinical variables indicated that age older than 65 is significantly associated to the onset of BC. Moreover, risk stratification based on the smoking status showed a slight positive association between smokers and BC incidence, confirming that the patient composition of the validation cohort accurately represents the general population and meets international statistics (Table 1) [8].

**Table 1:** Clinicopathological features of non-muscle-invasive bladder cancer (NMIBC) cases treated with Bacillus Calmette-Guérin (BCG).

Variables	*n* (%)	*p* value
Total	75	
Age at diagnosis, years (y)		
Median	72	
Range	42 - 95	0.0223
≤65 y	16 (21.3%)	
>65 y	59 (78.7%)	
Gender		1
Male	62 (82.6%)	
Female	13 (17.3%)	
Smoking status		0.071
Yes	50 (66.6%)	
No	24(32%)	
na	1 (1.4%)	
BMI		0.8307
Normal weight	36 (49.31%)	
Overweight	29 (39.73%)	
Obese	8(10.96%)	
BCG responsiveness		
Responder	28 (37.3%)	
Non-responder	47 (62.7%)	

Statistical significance has been calculated by Fisher’s exact test.

**Fig. 4:**
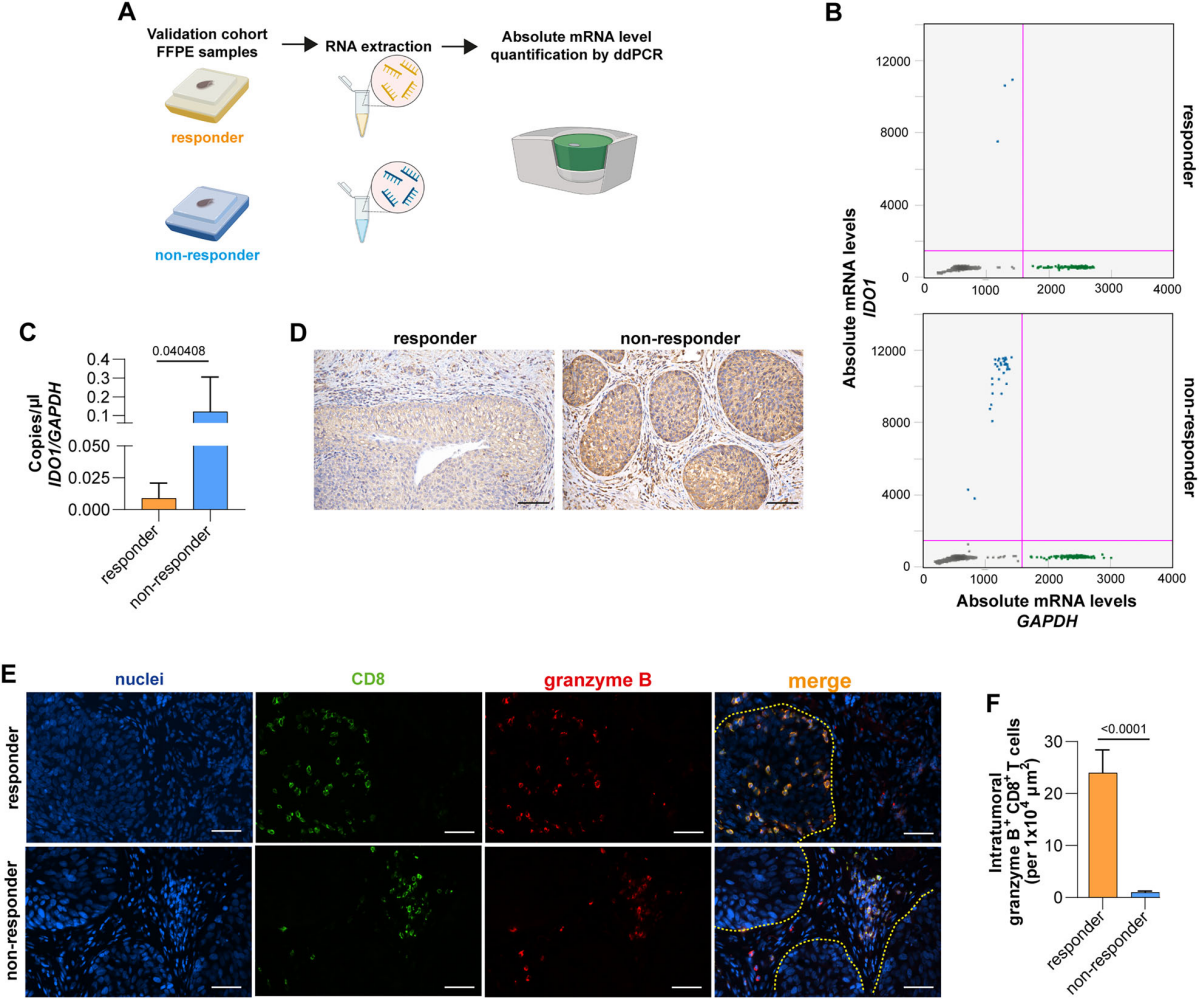
IDO1 is a predictive biomarker of BCG treatment response. **A** Workflow chart indicating the validation of IDO1 in our cohort of FFPE BC samples. **B** Representative droplet digital PCR (ddPCR) scatter plots showing positive droplets for IDO1 (blue) and GAPDH (green) used as housekeeping gene of FFPE samples of responder and non-responder BC patient. **C** Absolute mRNA levels (copies/µl) of IDO1 in responder and non-responder BC patients (*n* = 23). Data are represented as mean ± SD of three independent experiments. **D** Representative IHC analysis for IDO1 of patients as in (**C**). Scale bar is 100 µm. **E** Representative immunohistochemical analysis for CD8 and granzyme B in BC responder and non-responder BC patients. **F** Percentage of T cells positive for granzyme B and CD8 in responder and non-responder BC patients, as in (**E**), normalized to the tumor area analyzed.

To evaluate the potential prognostic value of IDO1 expression levels together with established predictors, we performed a gene analysis on RNA extracted from BC specimens obtained from BCG non-responder and responder patients and collected before treatment (Fig. 4A). As our retrospective cohort of patient samples were preserved as formalin-fixed paraffin-embedded (FFPE) tissues, a method that inherently affects RNA yield and integrity, we utilized droplet digital PCR (ddPCR) to increase the likelihood of successfully amplifying target transcripts. Compared to other PCR-based techniques, ddPCR provides superior sensitivity making it particularly effective for analyzing highly fragmented RNA [19–21]. ddPCR data revealed that IDO1 is more significantly expressed on unresponsive patients as compared to responsive patients (Fig. 4B, C), as also being confirmed at protein level by immunohistochemical analysis (Fig. 4D). Given its association with therapy failure, IDO1 could serve as a potential biomarker for predicting patient responses to BCG therapy, guiding personalized treatment strategies.

The interplay between IDO1 activity and immune suppression underlies the potential influence of this enzyme on the effectiveness of BCG therapy in patients with T1 HR NMIBC. This association becomes especially important when considering the crucial role of both the presence and spatial distribution of immune cells within the tumor microenvironment. Notably, high expression of *IDO1* was paralleled by a relevant increase of immune checkpoints (*PDCD1* (PD-1), *CD274* (PD-L1), *PDCD1LG2* (PD-L2), *LAG3* and *CTLA4*) level in our cohort of non-responder BC patients compared to responders (Fig. S3A).

IDO1’s ability to modulate the immune landscape may directly impact the efficacy of BCG treatment, potentially affecting patient outcomes and response rates. To confirm these findings, we subsequently validated T cell presence in our cohort of primary BC patients. Our analysis revealed a striking contrast between patients with favorable clinical outcomes and those who did not respond to BCG treatment. BC patients who experienced positive clinical outcomes demonstrated a greater infiltration of cytotoxic CD8^+^ T cells that were positive for granzyme B. This finding suggests a more robust and active immune response within the tumor microenvironment of these patients. Conversely, BCG non-responder patients exhibited a paucity of CD8^+^/granzyme^+^ T cells, with the few present being primarily confined to the stromal regions (Fig. 4E, F). This spatial constraint of cytotoxic T cells in non-responders may indicate a reduced ability of the immune system to effectively engage and eliminate tumor cells, potentially contributing to the poor treatment response observed in these patients.

## Discussion

Approximately one-third of NMIBC patients fail to respond to BCG therapy and over 50% experience recurrence or progression during long-term follow-up [22]. Radical cystectomy (RC) remains the standard treatment for T2 and very HR NMIBC, demonstrating significant efficacy in cancer eradication. However, many patients, particularly the elderly or those with significant comorbidities, are unsuitable candidates for RC, while others may be unwilling to undergo such extensive surgery. This underscores the urgent medical need for alternative therapeutic strategies that are both effective and less invasive.

Several therapeutic alternatives, including intravescical and immunotherapy-based approaches, have been explored, with pembrolizumab receiving FDA approval for BCG-resistant NMIBC [23–33]. Nevertheless the EAU guidelines consider treatments other than RC to be oncologically inferior in BCG-unresponsive patients. This underscores the ongoing challenge in managing these patients effectively. Thus, current research efforts are directed towards identifying early prognostic factors for BCG response [34]. Recent studies have investigated blood-based nutritional biomarkers, the prognostic nutritional index, and the urinary microbiome as potential predictive factors [35, 36], yet their clinical practice remains limited due to insufficient validation.

Our study aims to discover simple, reliable, and reproducible biomarkers for predicting early responses to BCG therapy in BC patients, enabling personalized treatment strategies. Early identification of non-responders could facilitate timely transitions to alternative therapies, such as early radical cystectomy, potentially preventing progression to MIBC and its associated complications. At present, there are no standardized models or biomarkers that reliably predict BCG response. The 2016 EORTC and CUETO risk scoring models primarily rely on clinicopathological features, which have limitations in predicting recurrence and lack additional biomarkers that could enhance predictive accuracy [37]. Genetic profiling has demonstrated that tumor genetics can significantly influence therapeutic response in BC. Whole-transcriptome analyses of NMIBC have led to clustering-based classifications and the identification of predictive signatures for disease progression [13, 38]. Despite extensive research efforts, genetic profiling studies in NMIBC have shown limited added value compared to standard clinical risk stratification.

Recent studies have identified promising immune suppressive genes associated with BCG treatment failure [39, 40]. Baek and Leem’s research has confirmed the value of multi-gene signatures in distinguishing NMIBC subtypes and suggested potential benefits for immunotherapy [41]. However, no immune markers with high sensitivity and specificity for predicting therapy response have been established. These studies are limited by an overrepresentation of BCG non-responders compared to real-world situations and the heterogeneity of data from various in vitro and in vivo studies.

Our data suggest that IDO1 may play a significant role in BC aggressiveness and response to BCG treatment. Since its discovery in the 1960s, inhibiting IDO1 has emerged as a promising approach to rejuvenate cancer immunosurveillance. IDO1 is primarily involved in regulating immune system responses and can be activated as negative feedback signaling by IFN-γ secreted by tumor-infiltrating lymphocytes, potentially contributing to tumor escape. Moreover, the analysis of differentially expressed genes here highlighted critical pathways and mechanisms that may underly the variability in patient responses to BCG therapy. Gene Set Enrichment Analysis (GSEA) based on transcriptomic data here revealed that the group of patients categorized as non-responders, who exhibited the worst overall survival and had previously been identified with high levels of IDO1 expression, also showed elevated expression of genes associated with epithelial-mesenchymal transition (EMT). Existing literature suggests that IDO1 promotes EMT in BC through the IL-6/STAT3/PD-L1 signaling pathway, enhancing the migratory and invasive potential of tumor cells [42]. Specifically, studies have shown that knockdown of IDO1 reduces N-cadherin and vimentin levels while increasing E-cadherin expression [43]. These findings highlight the significant role of EMT in BC aggressiveness and suggest that IDO1 could play a key role in regulating EMT marker expression in BC.

IDO1 has been reported to be expressed in both tumor and stromal cells [44]. IDO1 expression in stromal cells contributes to the establishment of a tumor-promoting microenvironment and support tumor progression. Stromal cells expressing IDO1 are associated with the establishment of an immunosuppressive microenvironment, creating conditions that favor the development of resistance to chemotherapy and immunotherapy [45, 46]. All these observations highlight the importance of studying and characterizing IDO1 in the context of tumor resistance to targeted therapy and chemotherapy. Moreover, the observations highlighted in this article underscore the importance of further investigating the role of IDO1 in future studies, not only as a marker in tumor cells but also within the context of the tumor microenvironment.

Various inhibitors are currently being tested in clinical trials, employing different strategies such as blocking IDO1’s enzymatic activity, reducing its expression, utilizing peptide vaccines, and targeting its effector modulators. While clinical studies indicate that IDO1 inhibitors alone have limited anti-tumor effects, their combination with other immunotherapies, such as checkpoint inhibitors, demonstrates synergistic potential to improve survival rates [47]. By understanding the molecular landscape of BCG therapy and the role of IDO1, we can pave the way for more effective treatment strategies in BC management, potentially leading to improved patient outcomes through personalized therapeutic approaches.

## Materials and methods

### Study populations

A total of 75 patients with NMBC were enrolled from the Unit of Urologic Oncology in “P. Giaccone” Hospital of Palermo (Number of ethical approval 11/2021, 15th December 2021). Retrospective studies were performed in accordance with the Declaration of Helsinki.

Classification of tumors has been performed in line with the TNM system of the Union for International Cancer Control (UICC) and the 2004 World Health Organization (WHO) grading system. All patients were treated with high-risk NMIBC criteria. Pre-BCG samples were obtained from primary incident tumor. Patients underwent to BCG instillation and routinely cystoscopy and cytologic urine control following EAU guidelines [48], six weekly instillations of BCG as induction therapy and successively maintenance therapy (every week for 3 weeks, and then up to 3 years after the start of the instillations). Cystoscopy evaluations were scheduled at 3 months post-BGC initiation, with further assessments based on response to treatment. BCG unresponsive patients included BCG-refractory tumors and those that develop T1/Ta HR recurrence within 6 months of completion of adequate BCG exposure or develop carcinoma in situ (CIS) within twelve months of completion of adequate BCG exposure, according to the latest EAU Guidelines [48]. The patients included in the study were categorized into two cohorts: responders (*n* = 28) and non-responders (*n* = 47) to bacillus Calmette–Guérin (BCG) therapy with a minimum follow-up of 2 years after first resection.

### Statistical analysis

The transcriptome profile (RNA-Seq analysis) of the training cohort has been retrieved by Robertson et al., 2020 (GSE154261) and comprises *n* = 73 naïve T1 HR BC patients treated with BCG therapy [13]. The training cohort population has been subsequently divided into clusters using k means 2. One cluster containing outlier samples has been excluded from the analysis and the second cluster comprising 65 samples, 36 non-responders and 29 responders, has been further analyzed.

Transcriptome profile of BC samples prior treatment have been analyzed. These two groups were analyzed for gene differentials. Out of the nearly 59,000 initial genes, 18,267 genes were retained following differential expression analysis using the R edgeR library. Among these, 1246 genes showed a p-value of less than 0.05, and 10 of these were coding genes with an absolute fold change of at least 2.

These differential genes are used for an Enrich Analysis using the EnrichR library in Ontology terms Biological Process, Molecular Function and Cellular Component. In addition, a GSEA was performed with the MSigDB library in the C2 class under Reactome level.

Kaplan-Meier curves of overall survival were generated by using the GSE32548, GSE48075, GSE31684 (*n* = 297) dataset comprising T1 HR BC patients treated with BCG. “High” and “Low” groups were defined by using the median expression of IDO1 gene in the patient cohort.

To identify ecotypes associated with BCG response, we applied the EcoTyper RNA-seq discovery framework using pre-defined settings on our discovery and validation cohort sourced from Robertson et al. (2020) (GSE154261) [13] (*n* = 6475).

Ecotype discovery was conducted using the EcoTyper framework developed by Luca et al. to identify and characterize cell states and ecosystem subtypes from bulk RNA-Seq data [17]. EcoTyper employs a community detection algorithm to uncover robust collaborative networks, referred to as ecosystem subtypes or ecotypes, within tissue samples. This analysis involved recovering TCGA RNA-Seq cohorts consisting of 10,485 samples.

All analyses were performed with R survival, survminer, and coxph libraries. Graphs were created by using the ggplot2 library.

### RNA extraction and droplet digital PCR

The RNA extraction from FFPE tumor tissue specimens was conducted using the RNeasy FFPE Kit (Qiagen). Subsequently, 2 μg of total RNA was retrotranscribed employing oligo(dT)-primer mix using Reliance Select cDNA Synthesis Kit (Bio-Rad). Specific gene expression (GEX) analysis was performed using 900 nM primers/250 nM probe (FAM) for *IDO1*, *PDCD1*, *PDCD1LG2* and *LAG3* genes, 900 nM primers/250 nM probe (HEX) for *CD274* and *CTLA4* genes, and 450 nM primers/125 nM probe (HEX) for GAPDH gene, with 1× of ddPCR supermix for probes (No-dUTP), using 500 ng of cDNA samples. Droplets were generated utilizing the QX200 Droplet Generator (Bio-Rad) and Droplet digital PCR (ddPCR- QX200 Droplet Reader) follow the protocol indicate in Turdo et al. [19].

### Immunohistochemistry and Immunofluorescence

FFPE bladder tissues were obtained from responder patients and non-responder BC patients treated with intravesical installations of Bacillus Calmette-Guérin (BCG).

Immunohistochemistry analysis was performed using a 5μm-thick paraffin-embedded section derived from BC samples and subsequently heated in a retrieval solution for antigen unmasking processes using the PT link system (Dako, Agilent Technologies, Santa Clara, CA, USA). Sections were permeabilized for 10 min on ice by using the 0.1% TRITON X-100 PBS and exposed overnight at 4 °C to IDO1 antibody (OTI2G4, mouse IgG1, Origine). Staining was revealed using a biotin-streptavidin-based reagent (Dako LSAB2 System-HRP) followed by detection with the DAB substrate chromogen (Dako). Mayer’s Hematoxylin (Lillie’s Modification) Histological Staining Reagent (Dako) has been used to counterstain nuclei.

For immunofluorescence analysis all slides were exposed overnight at 4 °C to primary antibodies against CD8 (C8/144B, mouse IgG1, Agilent) and Granzyme B (11F1, mouse IgG2a, Novocastra). Then, cells were labelled with secondary antibodies tagged with Alexa Fluor 488 (Invitrogen™) or Texas Red (ThermoFisher Scientific), and the nuclei were counterstained using DAPI stain (blue). Staining was analyzed using an ECLIPSE Ti2 invert microscope (Nikon).

## Supplementary information

Supplementary Figure Legends

Supplementary Table Legends

Supplementary Figure 1

Supplementary Figure 2

Supplementary Figure 3

Supplementary table 1

Supplementary table 2

## Data Availability

All datasets analyzed in this study are publicly available, as indicated in materials and methods section. All relevant raw data will be available on request from the corresponding author.
