# Supplementary material for: Heightened IDO1 levels predict Bacillus Calmette-Guèrin failure in high-risk non-muscle-invasive bladder cancer patients
Source: Cell Death Discov. 2025 Apr 26;11:203. doi: 10.1038/s41420-025-02489-7 (PMC12033280; doi:10.1038/s41420-025-02489-7)
Supplement: Supplementary file 1 — Supplementary Figure Legends [file 41420_2025_2489_MOESM1_ESM.doc]

**SUPPLEMENTARY FIGURE LEGENDS**

**Supplementary Figure 1.** (A) Clustering of responder and non responder BC patients cohort retrieved from the GSE154261 database (validation cohort) by k-2 means. (B) GSEA plot performed with the MSigDB library in the C2 class for epithelial mesenchymal transition, hallmark e2f targets, hallmark g2m checkpoint in responder an non responder BC patinets, (C) Bar plot showing top DEGs belonging to the Reactome signature. (D) GSEA plot performed with the MSigDB library in the C2 class for erbb2 regulates cell motility, erbb2 activates ptk6 signaling, reactome signaling by erbb2 in cancer in responder and non responder BC patients.

**Supplementary Figure 2.** (A-D) Cell state abundance patterns of B cells, CD4 T cells, CD8 T cells and dendritic cells in BC patients cohort of responder and non responder retrieved from GSE154261.

**Supplementary Figure 3.** (A) mRNA levels (copies/µl) of *PDCPD1* (PD-1), *CD274* (PD-L1), *PDCD1LG2* (PD-L2), *LAG3* and *CTLA4* in FFPE samples of responder and non responder BC patients (n=10). *GAPDH* was used as housekeeping control gene. Data are represented as mean  ±  SD of three independent experiments.
