# Supplementary material for: Heightened IDO1 levels predict Bacillus Calmette-Guèrin failure in high-risk non-muscle-invasive bladder cancer patients
Source: Cell Death Discov. 2025 Apr 26;11:203. doi: 10.1038/s41420-025-02489-7 (PMC12033280; doi:10.1038/s41420-025-02489-7)
Supplement: Supplementary file 2 — Supplementary Table Legends [file 41420_2025_2489_MOESM2_ESM.doc]

**SUPPLEMENTARY TABLE LEGENDS**

**Supplementary Table 1.** IDO1 mutational status of TCGA patients.

**Supplementary Table 2.** Clinicopathological features of non-muscle-invasive BC (NMIBC) cases treated with bacillus Calmette-Gérin (BCG) retrieved form the GSE32548, GSE48075 and GSE31684 (n=297) database.
