# Supplementary figures and images for: Heightened IDO1 levels predict Bacillus Calmette-Guèrin failure in high-risk non-muscle-invasive bladder cancer patients
Source: Cell Death Discov. 2025 Apr 26;11:203. doi: 10.1038/s41420-025-02489-7 (PMC12033280; doi:10.1038/s41420-025-02489-7)

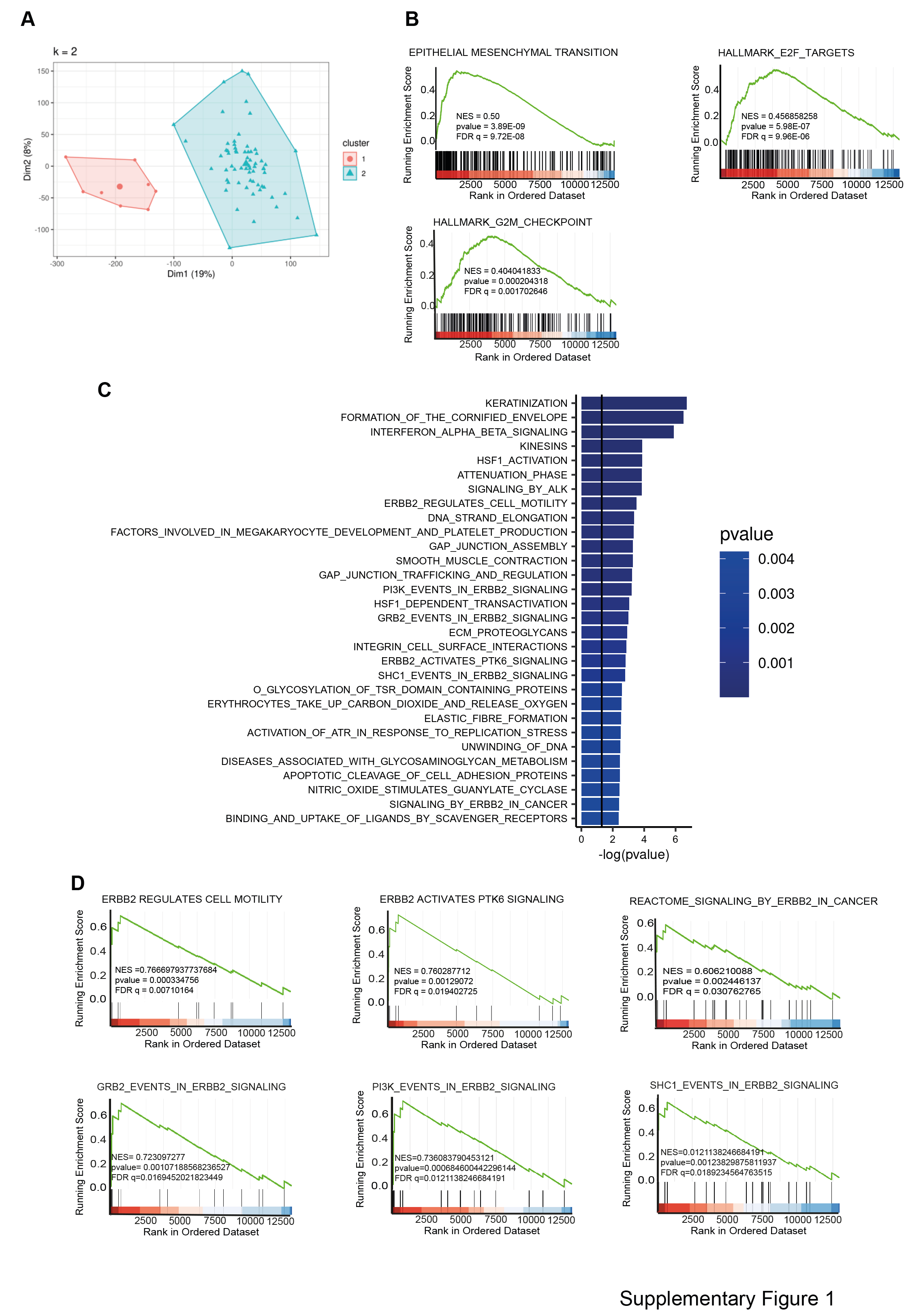

Supplement: Supplementary file 3 — Supplementary Figure 1 [file 41420_2025_2489_MOESM3_ESM.tif]

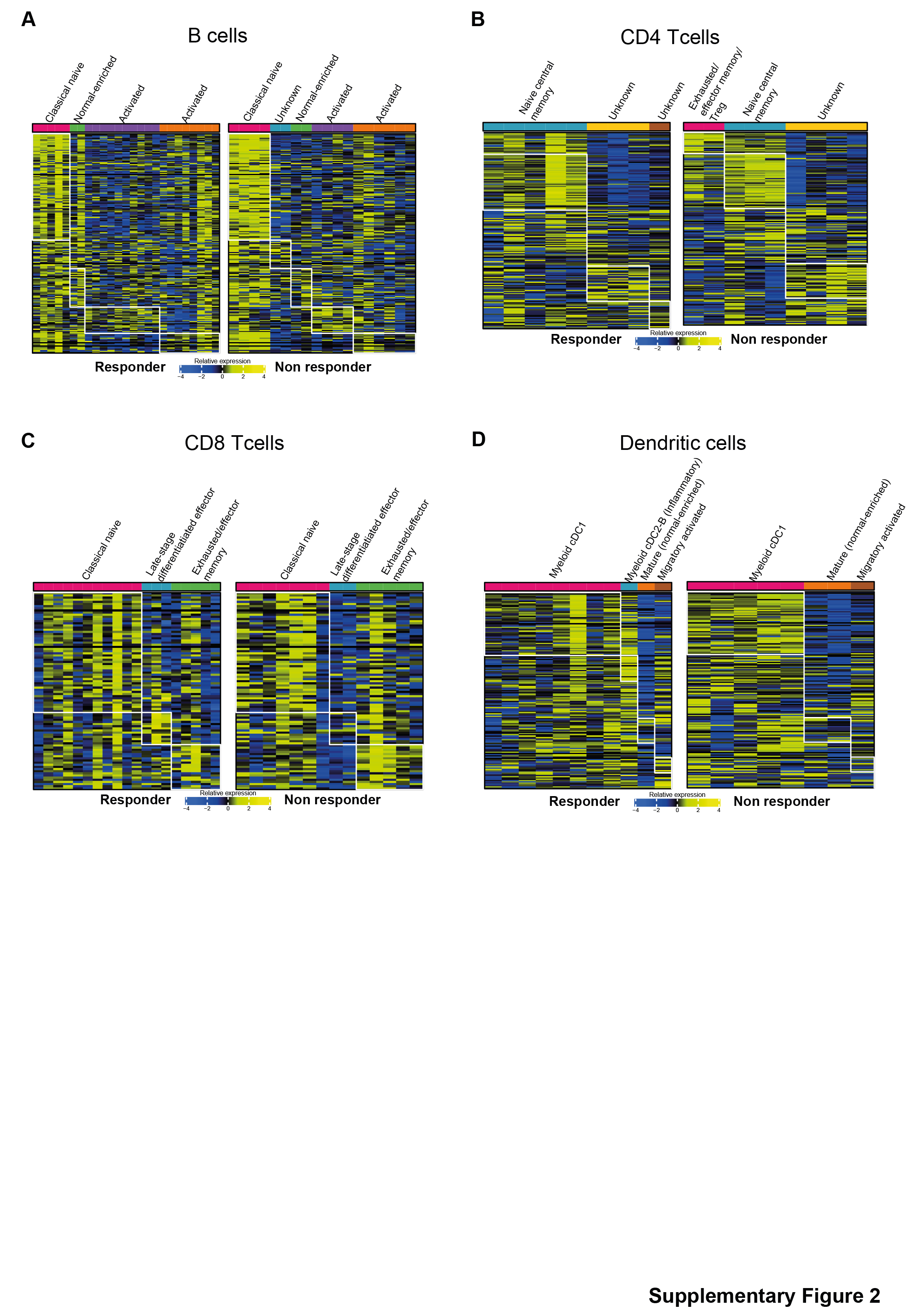

Supplement: Supplementary file 4 — Supplementary Figure 2 [file 41420_2025_2489_MOESM4_ESM.tif]

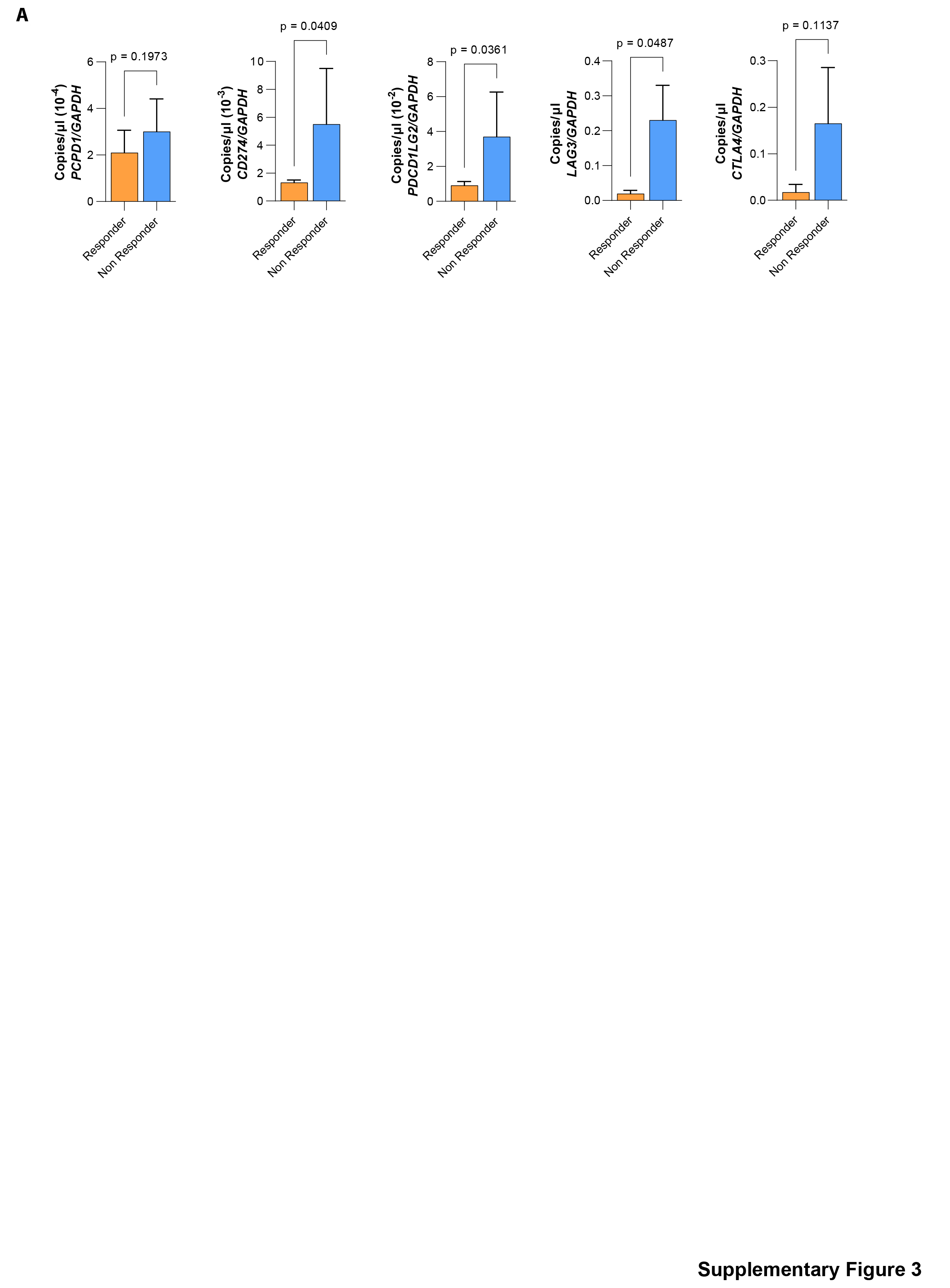

Supplement: Supplementary file 5 — Supplementary Figure 3 [file 41420_2025_2489_MOESM5_ESM.tif]
